# PReS-FINAL-2235: Overlap of homozygous TNFRSF1A R92Q mutation with MEVF E148Q mutation versus homozygous TNFRSF1A R92Q mutation: difference in clinical profile in two sisters from Oman

**DOI:** 10.1186/1546-0096-11-S2-P225

**Published:** 2013-12-05

**Authors:** R Abdwani, S Abrawi, E Ibraheem, K Al Thuhli

**Affiliations:** 1 Child Health, Sultan Qaboos University Hospital, Muscat, Oman; 2 Child Health, Royal Hospital, Muscat, Oman; 3 Genetics, Sultan Qaboos University Hospital, Muscat, Oman

## Introduction

Tumor necrosis factor receptor associated periodic syndrome (TRAPS) is a multifaceted auto inflammatory syndrome which was initially described in persons of Irish-Scottish ancestry and in ethnic groups of northern European descent. To date, more than 70 mutations have been described. Since then, it has been described in other ethnicities. There have been few reports of from Asia, 11 cases have been described from Japan, and only one case of TRAPS has been described in an Arab child from Israel (1-8).There are no cases of TRAPS that have been described from Gulf Arab states.

## Objectives

We hereby report 2 sisters with homozygous R92Q variant in the *TNFRSF1 *gene of Arabic origins from Oman with different clinical course, one which also had the E148Q mutation in *MEFV *gene.

## Methods

Detailed clinical description of two sisters including their family pedigree along with their laboratory investigations including measurement of TNF-α in both patients and their parents, in addition to sequencing of TNFRSF1A and MEFV.

## Results

12 yrs old girl presented at 18 months of age with episodes of high fever, lasting for 5 to 7 days occurring at monthly intervals. The attacks were associated with abdominal pain, vomiting, myalgias, arthralgia with occasional chest pains. Investigations during febrile episodes revealed anemia, leukocytosis, thrombocytosis and elevated inflammatory markers. Infectious, immunological, rheumatological and malignancy work up was negative. Sequencing of the *MEFV *gene revealed a heterozygous c.442G>C (E148Q) mutation resulting in the diagnosis of Familial Mediterranean Fever. She responded to colchicine for 4 years and to etanercpet for 2 years. Revision of the diagnosis was necessary due to change in clinical symptoms. The duration of fever was lasting up to 10-14 days with the occurrence of occasional periorbital swelling and redness. TRAPS was considered, and sequencing of the *TNFRSF1A *gene revealed homozygous R92Q variants. She was treated with anakinra with sustained dramatic clinical improvement for more than 12 months.

6 yrs old sister, presented at the age of 18 months with similar clinical episodes, but less severe in intensity and frequency. She had no significant clinical response to colchicines and showed transient response to etanercept lasting 3 months. She was also found to have homozygous R92Q variants in the *TNFRSF1A *gene. Anakinra was started with dramatic clinical improvement.

## Conclusion

R92Q is a nonstructural gene mutation with low disease penetrance resulting in a mild disease course in patients with TRAPS (9). We present 2 sisters with homozygous R92Q mutation presenting with different clinical disease course, a moderate and severe course which is unusual. In patients with atypical clinical features, an overlapping gene syndrome should be considered as in patient 1 who presented with a severe protracted clinical course not responding to conventional therapy. Perhaps the overlapping genes explain the difference in disease course. However, this postulation would contradict previous studies that found that the interaction between the MEFV gene and R92Q genes is minimal or non-existing (10).

## Disclosure of interest

None declared.

